# Palliative Management of Malignant Bowel Obstruction in Terminally Ill Patient

**DOI:** 10.4103/0973-1075.68403

**Published:** 2010

**Authors:** Darshit A Thaker, Bruce C Stafford, Luke S Gaffney

**Affiliations:** Medical Oncology Advanced Trainee, The Townsville Hospital, QLD, Australia; 1 Director, Palliative Care Unit, Redcliffe Hospital, QLD, Australia; 2 Internal Medicine Advanced Trainee, Fremantle Hospital, WA, Australia

**Keywords:** Malignant bowel obstruction, Complications of pancreatic cancer, Use of octreotide in palliative care

## Abstract

Mr. P was a 57-year-old man who presented with symptoms of bowel obstruction in the setting of a known metastatic pancreatic cancer. Diagnosis of malignant bowel obstruction was made clinically and radiologically and he was treated conservatively (non-operatively)with octreotide, metoclopromide and dexamethasone, which provided good control over symptoms and allowed him to have quality time with family until he died few weeks later with liver failure. Bowel obstruction in patients with abdominal malignancy requires careful assessment. The patient and family should always be involved in decision making. The ultimate goals of palliative care (symptom management, quality of life and dignity of death) should never be forgotten during decision making for any patient.

## INTRODUCTION

In palliative medicine, clinicians encounter patients having complications associated with advanced incurable cancers. The following is a case of not an uncommon clinical problem in palliative medicine but its management requires proper understanding of disease process, various treatment options and key concepts of palliation.

## CASE REPORT

Mr. P was diagnosed with unresectable locally advanced pancreatic cancer in April 2008. He received palliative chemotherapy with Gemcitabine for two months until he progressed on it with development of liver metastases. He was referred to our palliative care services for symptom management.

Mr. P’s abdominal pain was managed with controlled release oxycodone 30 mg bid. His bowels were kept regular with coloxyl + senna and Movicol. Two weeks later, he presented to the emergency department with symptoms of bowel obstruction. On arrival to our unit, Mr. P had continuous dull aching pain in his upper abdomen for 4-5 days, associated with abdominal distension, constipation and bilious vomiting. He was still able to mobilize without support and was independent in activities of daily living (Karnofsky score 60).

He was accompanied by his wife and two teenage children. He prepared his will and advanced health directives as he was well informed about prognosis. He wished to maintain quality of life for a few more weeks to sort out financial issues. He also expressed his wish to live in his home with family as long as possible but wanted to die in hospital.

On examination, he appeared weak and fatigued but he was not jaundiced. Abdomen was soft but distended with tenderness over epigastrium. There was no organomegaly and no signs of ascites. Bowel sounds were sparse on auscultation.

On admission to the unit, working diagnosis of malignant bowel obstruction was made. The patient was kept nil by mouth. Subcutaneous fluid infusion was started at the rate of 83 ml/h. Oral oxycodone was changed to equivalent dose of subcutaneous morphine infusion.

CT scan of abdomen with contrast showed large pancreatic mass and extensive hepatic metastases without intra or extra hepatic billiary obstruction. There was small and large bowel dilatation with tumor invasion suggesting obstructions at distal transverse colon and duodeno-jejunal flexure. There was also minimal ascites (150cc). Blood chemistry showed mildly elevated liver enzymes with normal bilirubin. Serum albumin was 30 g/L.

With confirmation of large and small bowel malignant obstruction, the patient was kept nil by mouth. Subcutaneous normal saline infusion was continued. Nasogastric tube placement was avoided for comfort, and the patient’s vomiting was controlled with regular subcutaneous metoclopramide 10 mg three times daily. Octreotide 100 mcg three times daily and dexamethasone 4 mg twice daily were started subcutaneously.

The surgeon offered duodenal stenting and distal colostomy or bypass surgery but explained to Mr. P the high risk of surgical mortality, postoperative morbidity and no improvement in overall survival. Considering the extent of the disease and the aggressive nature of his cancer, the overall prognosis was more likely weeks than months.

In family meeting, patient was informed about diagnosis, prognosis, surgical options with associated risk of postoperative mortality and morbidity and the pharmacological treatment but uncertainty of its results. He decided to continue conservative management and declined surgery. He wished to spend quality time with family and friends at home if symptoms could be controlled effectively.

In the next 2-3 days, symptomatically the patient started to improve. Abdominal distension lessened, vomiting ceased and the pain improved on the morphine infusion. His bowels started to open and the patient tolerated liquid and semisolid diet. Conservative treatment which included octreotide, dexamethasone and metoclopramide was continued for one week of inpatient hospital stay.

The patient was given the long acting somatostatin analogue (LANREOTIDE) intramuscularly to be repeated every 28 days. Subcutaneous dexamethasone and metoclopramide were changed to oral form. He was sent home on a continuous morphine infusion as there was a high likelihood of re-obstruction and worsening pain. He spent four weeks at home with his family and later readmitted with deep jaundice and abdominal distension, but no vomiting or constipation. He was still tolerating oral diet. The morphine infusion doses were modified for better analgesia. Ultrasound scan of the abdomen showed moderate to large volume ascites, further progression in liver metastases and intrahepatic biliary obstruction. Ascitic drainage was performed to reduce respiratory and abdominal discomfort with a good symptomatic response.

The patient’s overall condition deteriorated and he passed away five days later surrounded by his family and friends.

## DISCUSSION

Malignant bowel obstruction (MBO) is a common complication of various advanced malignancies particularly pancreatic, colorectal and peritoneal carcinomatosis of ovarian cancer.

It is essential to determine the underlying cause of bowel obstruction, as non malignant causes like intra-abdominal bands or adhesions due to prior surgery, post radiation fibrosis or fecal impaction secondary to ongoing morphine use will require definitive treatment. In one study, 48% of bowel obstruction in colorectal cancer had non malignant aetiologies.[1]

The malignant causes are either mechanical obstruction due to external compression/intraluminal tumor growth or functional obstruction due to tumor infiltration of bowel wall muscle or nerves leading to paralytic ileus.[2]

Differential diagnosis in this case would include opioid-induced constipation with fecal impaction. A plain abdominal X-ray is a simple and valuable investigation to ascertain obstruction and also presence of constipation. But CT scan is required to determine the cause of obstruction. CT scan has a reported sensitivity of 93% and specificity of 100% in determining the cause of bowel obstruction. Moreover, Gastrogaffin used as a contrast agent can reduce luminal edema and resolve partial obstruction.[3]

Symptoms occasionally suggest the location of obstruction particularly when it is at a single level. Proximal obstruction usually presents predominantly with bilious vomiting and periumbilical pain while distal obstruction typically is characterized by abdominal distension and constipation.[2]

Treatment of the patient with MBO is a challenging clinical scenario as decision making needs delicate balance between pros and cons of intervention. It is influenced by the level of obstruction, clinical stage of cancer, overall prognosis, presence of ascites as well as patient’s performance status.[2]

Palliative surgery, which includes resection, stoma formation and endoscopic stent placement, is a reasonable option for selected patients with MBO as about one third will have prolonged postoperative survival with acceptable, treatment-related morbidity. Gastric outlet obstruction can be treated with gastric venting via PEG tube insertion which reduces intractable nausea and vomiting.[4]

Self-expanding metallic stent placement is an effective non invasive procedure. It provides good palliation for unresectable advanced tumor causing obstruction not only in proximal bowel but also in left-side of colon.[5]

YAG laser photoablation is also an option in patients with Malignant rectosigmoid obstruction. It provides reasonable long term symptom relief and controls bleeding. With stenting, it is an alternative to colostomy without surgical morbidity. The major drawbacks are requirement of multiple sessions and risk of colonic perforation.[6]

Guidelines for surgery in such patients include life expectancy of greater than two months, good functional status and single site of obstruction. Patients are considered as poor candidates for surgery if there is a history of previous palliative surgery, a history of inoperability, presence of ascites, peritoneal carcinomatosis and poor functional status. An ascites volume of 100 ml or more is a predictor of poor outcome.[7] TSF (tolerating solid food) at discharge is a useful predictor of continued palliation for most patients.

Considering young age, good performance status and no history of previous surgery, palliative surgery could have been considered for Mr. P. The negative predictors of surgical outcome were multiple sites of obstruction, presence of ascites, presence of extensive liver metastases and pleural effusion, failure of chemotherapy, aggressive nature of cancer and prognosis of less than two months.

Medical treatment should begin with treatment of dehydration as patients with MBO tend to be dehydrated early due to accumulation of water and electrolytes in intestine and poor fluid intake due nausea and vomiting. Preferred means of managing dehydration in this situation is instituting hypodermoclysis, rather than intravenous fluids. This route for hydration has been established as a safe, comfortable and effective option for providing parenteral fluids to patients with MBO.[8]

Pharmacological treatment includes a combination of analgesics, antiemetics, antisecretory drugs and steroids. Early introduction of pharmacological treatment can reduce symptoms, reverse malignant bowel obstruction and provide better quality of life and quality of death.[9]

The administration of analgesics, mainly continuous subcutaneous infusion of morphine should alleviate background abdominal pain. If colic persists despite the use of an opioid, anticholinergic drugs such as hyoscine butylbromide 40-120 mg/24h subcutaneously should also be administered in association with opioid.[10]

Management of nausea and vomiting can be achieved by a cautious trial of a prokinetic agent such as metoclopramide 30 mg-60 mg/24h subcutaneously if the patient is passing flatus. It may help resolve incomplete obstruction. It is also the drug of choice in patients with functional bowel obstruction but not recommended in the presence of complete bowel obstruction and in gastric outlet obstruction. When metoclopramide is not helpful, cyclizine 100-150 mg/24 h or haloperidol 5-10mg/24h subcutaneously is also recommended. If the patient is experiencing colic with nausea, hyoscine butylbromide should be considered due to its antisecretory and antispasmodic effects. When all else fails, some centers recommend subcutaneous methotrimeprazine (phenothiazine derivative) 25-150mg/24hr. Sedation is the major side effect of this drug. Some patients may benefit from a 5HT3 receptor antagonist (ondansetron), as bowel distension causes the release of serotonin/5HT from enterochromaffin cells in the bowel.[11]

The somatostatin analogue (octreotide) has similar effects as hyoscine to reduce gastrointestinal secretions but without anticholinergic effects. Octreotide also inhibits release of gastrointestinal hormones, reduces gastric acid secretions, slows intestinal motility, decreases bile flow and reduces splanchnic blood flow. It may relieve partial bowel obstruction secondary to mechanical causes as it reduces the hypertensive state within the lumen of the gut. It is used in the dose range of 300-600 mcg/24h subcutaneously.[12]

Corticosteroid has controversial role in malignant bowel obstruction. It may help by reducing peritumoral inflammatory edema if given in appropriate doses. As per Cochrane review 2008, “there is a trend for evidence that dose range 6 to 16 mg dexamethasone given intravenously/subcutaneously may bring about the resolution of bowel obstruction. Equally, the incidence of side effects in all the included studies is extremely low. Corticosteroids do not seem to affect the length of survival of these patients.”[13]

Finally, palliative radiotherapy at the site of obstruction is a consideration, though as yet there is little information on outcome of this approach.[14] In this case, it was not an option due to multiple sites of obstruction.

The combination of metoclopramide, octreotide and steroids given in appropriate doses provides an excellent palliative result. This approach is also supported by a study published in “Supportive Care in Cancer” (1996, vol 4), in which above mentioned combination and single dose of amidotrizoate relieved MBO, improved intestinal transit within 1-5 days and maintained bowel patency until death in most of the patients.[15]

With a diagnosis of MBO at multiple sites with aggressive pancreatic cancer, Mr. P had a prognosis of a few weeks. Surgery would not have increased survival but might have increased morbidity. He responded very well to pharmacological treatment of MBO and managed to spend his last few weeks at home with family with reasonable quality of life.

Though, in selected groups of patients, benefit of surgery over medical management is seen - mainly those having single obstruction, response to chemotherapy, absence of ascites or extensive metastases, and in relatively young patients with good performance status. This group of patients should not be missed. When bowel obstruction is due to benign causes like a band or adhesions, the surgical approach is the treatment of choice.
